# A Stress-Responsive Nuclear Factor, GLDI-8, Mediates Mitochondrial Stress Responses to ETC Dysfunction

**DOI:** 10.3390/ijms27104387

**Published:** 2026-05-14

**Authors:** Yung Wu, Hongyun Tang

**Affiliations:** 1 College of Life Sciences, Zhejiang University, Hangzhou 310058, China; 2 State Key Laboratory of Gene Expression, School of Life Sciences, Westlake University, Hangzhou 310024, China; 3 Westlake Laboratory of Life Sciences and Biomedicine, Hangzhou 310024, China

**Keywords:** *Caenorhabditis elegans*, electron transport chain dysfunction, mitochondrial unfolded protein response, GLDI-8

## Abstract

Mitochondrial electron transport chain (ETC) impairment triggers mitochondrial unfolded protein response (UPR^mt^) that promotes mitochondrial homeostasis, yet the nuclear factors that mediate these responses remain incompletely defined. Here, we identify GLDI-8 as a nuclear factor required for robust activation of the *hsp-6p::gfp* UPR^mt^ reporter induced by ETC dysfunction in *Caenorhabditis elegans*. Depletion of *gldi-8* markedly compromises mitochondrial stress-induced *hsp-6p::gfp* reporter activation, and transgenic rescue restores the response, supporting a specific requirement for GLDI-8 in this pathway. Mitochondrial stress promotes nuclear accumulation of GLDI-8; however, a GLDI-8 transcriptional (promoter) reporter shows no detectable induction under the same conditions, suggesting that regulation occurs at the post-transcriptional level. Genetic analysis further shows that stress-induced nuclear translocation of GLDI-8 is not abolished by *atfs-1* knockdown, and GLDI-8 is dispensable for DVE-1 nuclear translocation under mitochondrial stress. Together, these findings establish GLDI-8 as a mitochondrial stress-responsive nuclear factor that contributes to ETC impairment–induced transcriptional responses and adds to the complex regulatory network underlying the UPR^mt^.

## 1. Introduction

Mitochondria are essential organelles that serve as the primary site for cellular energy production through oxidative phosphorylation, mediated by the electron transport chain (ETC) [1,2]. However, impairments in ETC function, arising from genetic mutations, environmental toxins, or aging, can lead to the accumulation of misfolded proteins and reactive oxygen species, compromising mitochondrial homeostasis and contributing to various pathologies, including neurodegenerative diseases, metabolic disorders, and cancer [2,3,4]. To cope with such perturbations, eukaryotic cells have evolved adaptive mitochondria-to-nucleus signaling pathways, among which the mitochondrial unfolded protein response (UPR^mt^) is a well-characterized mechanism that reprograms nuclear gene expression to promote mitochondrial recovery and homeostasis [5,6,7,8,9,10,11,12].

In the model organism *C. elegans*, UPR^mt^ involves the transcription factors ATFS-1 and DVE-1, which translocate to the nucleus upon mitochondrial stress to activate protective genes [9,13,14,15,16]. In addition to transcription factors, UPR^mt^ also relies extensively on epigenetic and chromatin-based regulation [8]. Mitochondrial stress induces chromatin reorganization through the histone methyltransferase MET-2 and its cofactor LIN-65 [17], while the conserved histone demethylases JMJD-1.2 and JMJD-3.1 are required for stress-induced longevity and UPR^mt^ activation and can promote this response when overexpressed [18]. The acetyltransferase CBP-1 acts downstream of JMJD-1.2 and JMJD-3.1 and upstream of ATFS-1 to promote transcriptional activation of UPR^mt^ genes [19], whereas the deacetylase HDA-1 cooperates with DVE-1 to regulate mitochondrial stress-responsive, immune, and metabolic gene expression [20]. Metabolic state adds an additional layer to UPR^mt^-associated chromatin reorganization, as impaired TCA cycle activity lowers citrate and acetyl-CoA levels, promoting nuclear accumulation of NuRD and DVE-1 and thereby coupling mitochondrial metabolic stress to histone deacetylation and chromatin remodeling [21,22]. Together, these findings indicate that mitochondrial stress-responsive transcription depends on multilayered chromatin remodeling and transcriptional pathways.

Accumulating evidence suggests that mitochondrial perturbations do not trigger a single uniform transcriptional program. Rather, distinct types of mitochondrial dysfunction can generate overlapping but non-identical nuclear responses [6,8,13,15,17,18], indicating that mito-nuclear communication is modular and context-dependent. In particular, whether additional mitochondrial stress-responsive transcription factors contribute to UPR^mt^ remains unclear.

A common principle in organelle stress signaling is that regulatory factors can be activated not only through transcriptional induction but also through post-transcriptional mechanisms such as altered protein trafficking and subcellular localization [8,13,23,24]. In the mitochondrial stress response, such regulation offers a rapid means to couple organelle dysfunction to nuclear transcriptional adaptation. ATFS-1 provides a clear example of this logic, as its accumulation in the nucleus is controlled by mitochondrial protein import efficiency [13]. However, whether mitochondrial stress also mobilizes additional stress-responsive nuclear factors through mechanisms distinct from this canonical pathway remains incompletely understood.

In this study, we sought to determine whether additional stress-responsive nuclear factors contribute to ETC dysfunction-associated mitochondrial stress responses in *C. elegans*. We identify GLDI-8 as a factor required for robust activation of the *hsp-6p::gfp* UPR^mt^ reporter under ETC dysfunction and further examine its mitochondrial stress-associated nuclear accumulation and genetic relationship with established UPR^mt^ regulators. These findings provide a basis for understanding how GLDI-8 contributes to the regulatory network engaged during mitochondrial stress.

## 2. Results

### 2.1. gldi-8 Is Required for Robust Activation of the hsp-6p::gfp UPR^mt^ Reporter Under ETC Dysfunction

**Figure 1:**
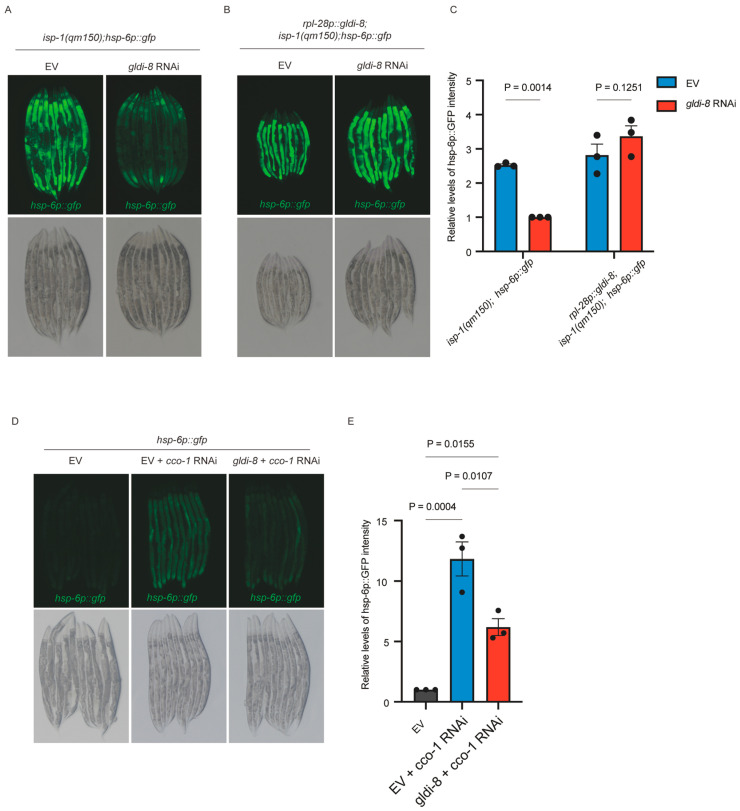
*gldi-8* is required for robust activation of the *hsp-6p::gfp* UPR^mt^ reporter under ETC dysfunction. (**A**) Representative fluorescence images showing that RNAi-mediated knockdown of *gldi-8* suppresses the upregulation of the *hsp-6p::gfp* UPR^mt^ reporter in *isp-1(qm150)* mutants. (**B**) Transgenic expression of GLDI-8::FLAG under the ubiquitous *rpl-28* promoter restores *hsp-6p::gfp* upregulation in animals treated with *gldi-8* RNAi, as assessed by *hsp-6p::gfp* expression. (**C**) Quantification of *hsp-6p::gfp* fluorescence intensity in the conditions shown in (**A**,**B**). *n* = 30 worms for each condition from 3 independent biological experiments. Data are presented as mean ± SEM, *p* values were calculated by two-way ANOVA followed by an uncorrected Fisher’s LSD test. (**D**) Representative fluorescence images showing that *gldi-8* RNAi suppresses *cco-1* RNAi-induced upregulation of *hsp-6p::gfp*. (**E**) Quantification of *hsp-6p::gfp* fluorescence intensity in the conditions shown in (**D**). *n* = 30 worms for each condition from 3 independent biological experiments. Data are presented as mean ± SEM, *p* values were calculated by one-way ANOVA with Tukey’s multiple-comparison test.

To identify nuclear factors that contribute to mitochondrial stress-induced transcriptional responses, we performed an RNAi-based screen using a transcription factor sublibrary assembled from the Ahringer and Vidal RNAi collections in worms carrying the constitutively active UPR^mt^ reporter background *isp-1(qm150)*; *hsp-6p::gfp*. In this genetic context, impaired electron transport chain (ETC) activity causes robust activation of the *hsp-6p::gfp* reporter [25,26], thereby creating a sensitized background for identifying regulators required for full UPR^mt^ induction. This screen identified *gldi-8* as a reproducible positive regulator of *hsp-6* reporter activation (Figure 1A,C). Specifically, RNAi-mediated depletion of *gldi-8* markedly reduced the elevated *hsp-6p::gfp* fluorescence observed in *isp-1(qm150)* animals (Figure 1A,C), indicating that *gldi-8* is required for robust activation of the *hsp-6p::gfp* UPR^mt^ reporter.

Because *gldi-8* loss is lethal, we next asked whether the suppression caused by *gldi-8* RNAi reflected a specific requirement for *gldi-8*, rather than a nonspecific consequence of RNAi treatment. To address this, we generated a rescue transgene expressing a codon-optimized, RNAi-resistant *gldi-8* coding sequence under the control of the ubiquitous *rpl-28* promoter. Transgenic expression of *rpl-28p::gldi-8::flag* restored *hsp-6p::gfp* activation in *isp-1(qm150)* animals subjected to *gldi-8* RNAi (Figure 1B,C), supporting the specificity of the RNAi phenotype and demonstrating that GLDI-8 is functionally required for this stress response output.

We then tested whether this requirement was specific to the *isp-1* mutant background or instead reflected a broader role in ETC dysfunction-induced *hsp-6p::gfp* reporter activation. To this end, mitochondrial stress was induced independently by RNAi-mediated knockdown of *cco-1*, a core ETC component. As expected, *cco-1* RNAi strongly induced the *hsp-6p::gfp* reporter, whereas simultaneous depletion of *gldi-8* markedly suppressed this induction (Figure 1D,E). Thus, *gldi-8* is required for *hsp-6p::gfp* reporter activation across two distinct paradigms of ETC dysfunction, one genetic and one RNAi-based.

### 2.2. Mitochondrial Stress Promotes Nuclear Accumulation of GLDI-8

**Figure 2:**
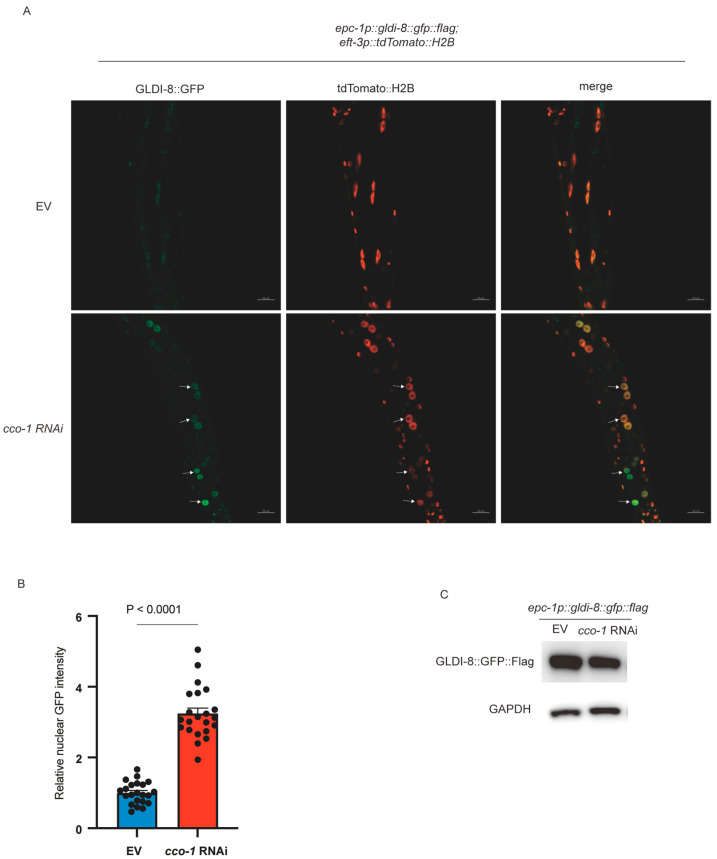
Mitochondrial stress induces nuclear accumulation of GLDI-8. (**A**) Representative confocal images of animals expressing *epc-1p::gldi-8::gfp::flag* show that GLDI-8 exhibits weak nuclear localization in intestinal cells under basal conditions but accumulates prominently in nuclei upon mitochondrial stress induced by *cco-1* RNAi. tdTomato::H2B was used as a nuclear marker. Arrows indicate nuclear-localized GLDI-8::GFP. Scale bar, 20 μm. (**B**) Quantification of nuclear GFP intensity in the conditions shown in (**A**). *n* = 22 worms for each condition. Data are presented as mean ± SEM. *p* values were calculated using an unpaired *t* test with Welch’s correction. (**C**) Western blot analysis of total GLDI-8 protein levels in animals exposed to EV or *cco-1* RNAi, showing no substantial change in overall GLDI-8 protein abundance under mitochondrial stress conditions.

Having established that *gldi-8* is required for *hsp-6p::gfp* upregulation, we next asked how GLDI-8 itself responds to mitochondrial stress. Because UPR^mt^ involves stress-dependent changes in nuclear gene regulation, and several regulators, including ATFS-1 and DVE-1, undergo nuclear accumulation upon mitochondrial stress, we examined the subcellular localization of GLDI-8 under basal and mitochondrial stress conditions. To do so, we generated an integrated translational reporter in which *gldi-8::gfp* was expressed under the control of the *epc-1* upstream region (2.3 kb). This promoter fragment was chosen because *epc-1* and *gldi-8* reside in the same operon. Intestinal nuclei were identified using *tdTomato::H2B* as a nuclear marker. Under control conditions, GLDI-8::GFP showed only weak nuclear localization in intestinal cells (Figure 2A). In contrast, upon *cco-1* RNAi, GLDI-8 accumulated prominently in nuclei (Figure 2A,B), indicating that mitochondrial stress promotes nuclear accumulation of GLDI-8. This mitochondrial stress-dependent nuclear accumulation indicates that GLDI-8 functions as a stress-responsive nuclear factor, rather than as a constitutively nuclear transcriptional regulator.

**Figure 3:**
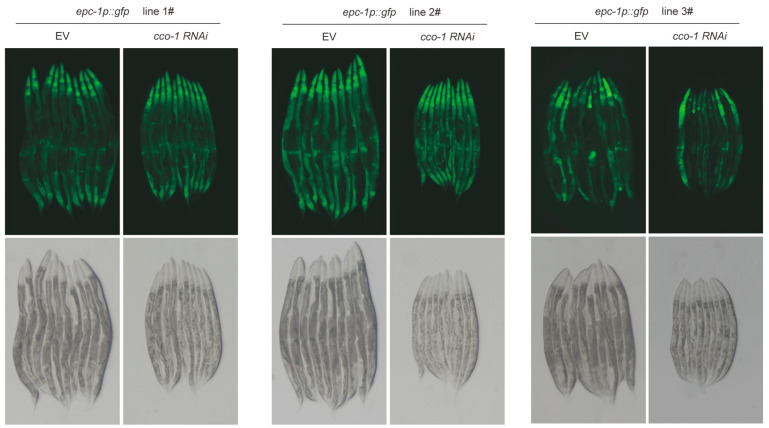
*gldi-8* promoter reporter is not detectably induced by mitochondrial stress. Representative fluorescence images of integrated *epc-1p::gfp* reporter lines under control and *cco-1* RNAi conditions. All three lines showed similar expression patterns and no obvious increase in GFP fluorescence upon mitochondrial stress.

We next asked whether the increased nuclear GLDI-8 signal might simply reflect increased overall expression. To address this, we first assessed total GLDI-8 protein levels by Western blot analysis and found that GLDI-8 protein abundance did not change substantially under mitochondrial stress conditions (Figure 2C). We then examined three independent integrated transgenic lines carrying an *epc-1p::gfp* transcriptional reporter. All three lines exhibited similar GFP expression patterns and showed no detectable increase in reporter fluorescence upon *cco-1* RNAi treatment (Figure 3). Together, these data suggest that mitochondrial stress regulates GLDI-8 primarily at the post-transcriptional level. Rather than being induced through increased gene expression, GLDI-8 appears to be mobilized through stress-dependent subcellular translocation into the nucleus.

### 2.3. GLDI-8 Nuclear Accumulation Under Mitochondrial Stress Is Not Abolished by atfs-1 Knockdown

We next sought to position GLDI-8 relative to the canonical ATFS-1 pathway. ATFS-1 is a central regulator of the *C. elegans* UPR^mt^ and is thought to sense mitochondrial stress by sensing changes in mitochondrial protein import efficiency. Under basal conditions, ATFS-1 is imported into mitochondria and degraded by the matrix protease Lon, whereas mitochondrial stress impairs import and thereby allows ATFS-1 to accumulate in the nucleus and activate stress-responsive genes [13]. If GLDI-8 nuclear accumulation were downstream of this canonical import-surveillance pathway, one possibility would be that depletion of *atfs-1* might impair or abolish stress-induced nuclear localization of GLDI-8.

**Figure 4:**
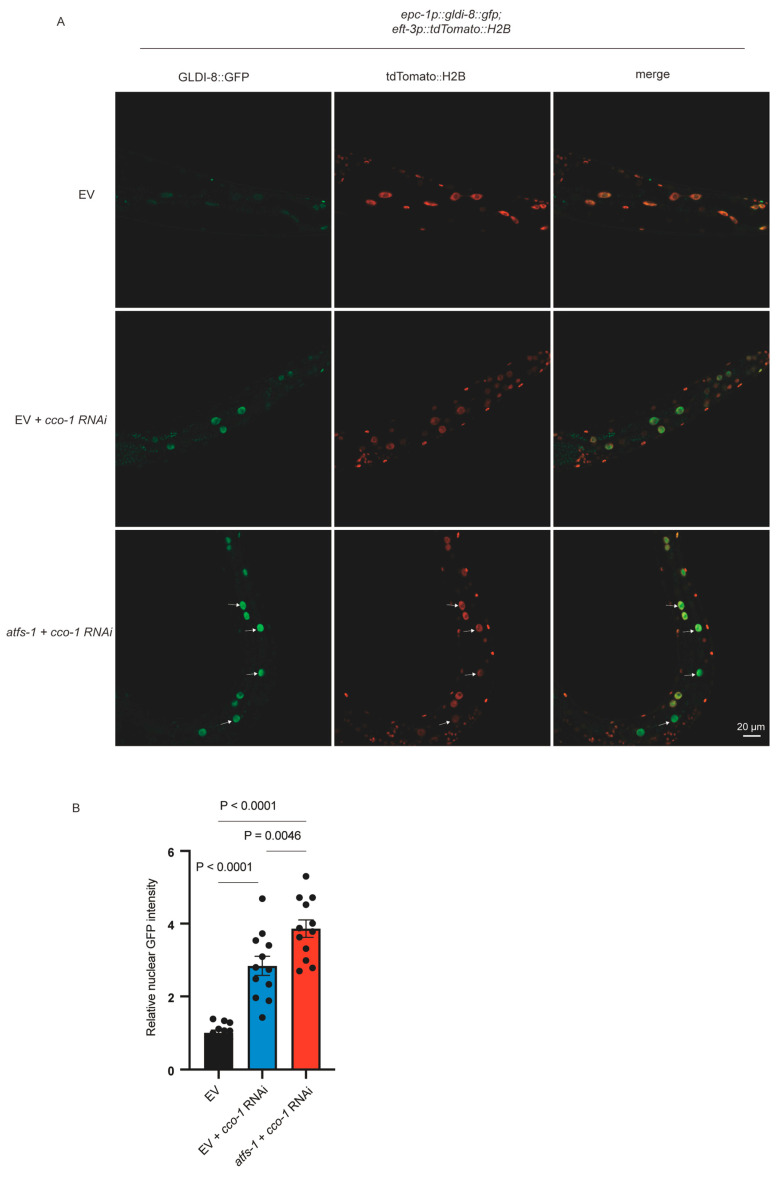
ATFS-1 is not required for mitochondrial stress-induced nuclear accumulation of GLDI-8. (**A**) Representative images showing that *atfs-1* RNAi enhanced mitochondrial stress-induced nuclear accumulation of GLDI-8 in animals expressing *epc-1p::gldi-8::gfp*. Arrows indicate nuclear-localized GLDI-8::GFP. Scale bar, 20 μm. (**B**) Quantification of nuclear GFP intensity in the conditions shown in (**A**). *n* = 12 worms for each condition. Data are presented as mean ± SEM, *p* values were calculated by one-way ANOVA with Tukey’s multiple-comparison test. The *cco-1* RNAi conditions were performed in a double-RNAi format (1:1 bacterial mixing), which may contribute to differences in the apparent extent of GLDI-8 nuclear accumulation compared with Figure 2.

However, RNAi-mediated knockdown of *atfs-1* did not abolish *cco-1* RNAi-induced nuclear accumulation of GLDI-8::GFP (Figure 4A,B). Instead, *atfs-1* RNAi markedly enhanced GLDI-8 nuclear localization (Figure 4A,B). This result indicates that GLDI-8 nuclear translocation does not require ATFS-1 and therefore is unlikely to represent a simple downstream consequence of the canonical ATFS-1-dependent protein import sensing pathway.

The enhanced nuclear accumulation of GLDI-8 upon *atfs-1* depletion further supports the view that ATFS-1 is not required for the nuclear accumulation of GLDI-8 under mitochondrial stress. Although the mechanism underlying this enhancement remains unclear, the mechanistic relationship between GLDI-8 and ATFS-1 in UPR^mt^ remains to be determined.

### 2.4. gldi-8 Is Dispensable for DVE-1 Nuclear Translocation upon Mitochondrial Stress

To further define the position of GLDI-8 within the mitochondrial stress signaling hierarchy, we examined its relationship with DVE-1, another key nuclear regulator of the UPR^mt^. DVE-1 undergoes nuclear accumulation during mitochondrial stress and has been implicated in transcriptional and chromatin-based remodeling of the stress response [15,22]. If GLDI-8 acted upstream of DVE-1, then depletion of *gldi-8* would be expected to impair DVE-1 nuclear accumulation under stress conditions.

**Figure 5:**
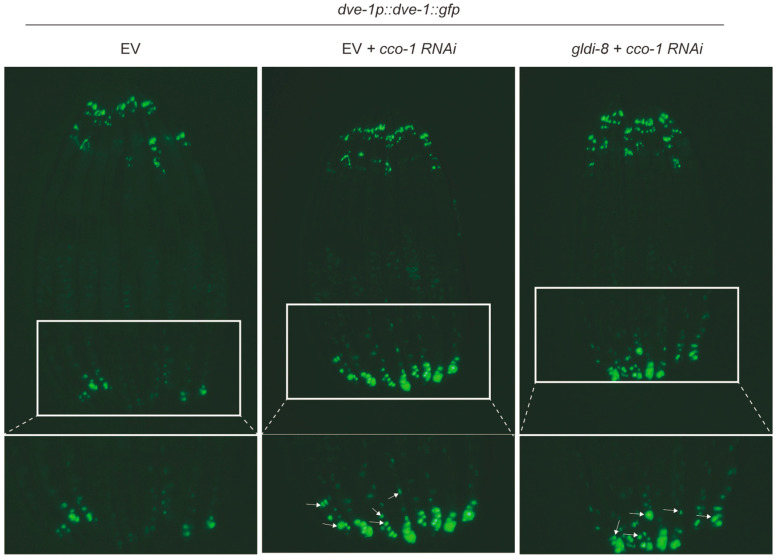
*gldi-8* is not required for DVE-1 nuclear translocation upon mitochondrial stress. Representative images showing that RNAi-mediated knockdown of *gldi-8* does not impair mitochondrial stress-induced nuclear accumulation of DVE-1. Arrows indicate nuclear-localized DVE-1::GFP.

To test this possibility, we examined DVE-1 localization in animals subjected to *gldi-8* RNAi during mitochondrial stress. In contrast to its strong effect on *hsp-6p::gfp* induction, depletion of *gldi-8* did not prevent the stress-induced nuclear accumulation of DVE-1 (Figure 5). Thus, *gldi-8* is dispensable for this relocalization event. GLDI-8 is unlikely to function upstream of DVE-1 nuclear translocation as an initiating regulator of this step. Instead, it may act in parallel to DVE-1, or downstream of nuclear translocation events, to promote the full transcriptional output of UPR^mt^ signaling.

## 3. Discussion

In this study, we identify GLDI-8 as a stress-responsive nuclear factor essential for UPR^mt^ activation in response to ETC dysfunction in *C. elegans*. GLDI-8 depletion markedly compromises activation of the UPR^mt^ reporter, and transgenic rescue restores the response, supporting a specific requirement for GLDI-8 in this stress context. We further show that mitochondrial stress promotes nuclear accumulation of GLDI-8. Notably, a *gldi-8* promoter reporter is not detectably induced by mitochondrial stress, suggesting that GLDI-8 is regulated primarily at a post-transcriptional level. Finally, *atfs-1* knockdown does not abolish stress-induced nuclear accumulation of GLDI-8, and *gldi-8* depletion does not prevent DVE-1 nuclear accumulation, indicating that GLDI-8 is not simply downstream of canonical ATFS-1 regulation and is dispensable for DVE-1 nuclear translocation under mitochondrial stress.

ATFS-1 is thought to monitor mitochondrial dysfunction through changes in protein import efficiency [13], whereas mitochondrial stress perturbs multiple aspects of mitochondrial physiology, including reactive oxygen species, membrane potential, metabolic output, and proteostasis. The independence of GLDI-8 nuclear accumulation from ATFS-1 raises the possibility that GLDI-8 is engaged by an upstream signaling pathway distinct from the canonical mitochondrial protein import surveillance mechanism. Our findings therefore suggest that mitochondrial stress may be sensed not through a single unified mechanism, but instead through multiple parallel inputs that detect distinct features of mitochondrial stress. In this framework, GLDI-8 may respond to a stress signal that is different from the signal that controls ATFS-1, thereby allowing cells to activate signal-specific protective programs.

Despite these insights, several limitations warrant consideration. Our study focuses specifically on ETC dysfunction, and it remains unclear whether GLDI-8 also participates in responses to other forms of mitochondrial stress, such as proteotoxic stress from unfolded proteins or oxidative damage. In addition, although our data identify GLDI-8 as a factor required for robust activation of the *hsp-6p::gfp* UPR^mt^ reporter, the broader transcriptional program regulated by GLDI-8 remains undefined. Future analysis of additional endogenous UPR^mt^ target genes will be important to determine whether GLDI-8 controls a broad mitochondrial stress transcriptional program or a more selective subset of genes. More comprehensive transcriptomic approaches, such as RNA-seq, will further help define the transcriptional scope of GLDI-8-dependent regulation. In parallel, identifying the direct genomic targets of GLDI-8, for example, by ChIP-seq or related approaches, will be important for clarifying how GLDI-8 contributes to mitochondrial stress-responsive transcription.

In summary, our findings identify GLDI-8 as a previously unrecognized component of the mitochondrial stress response and suggest that it responds to mitochondrial stress in a manner that is not simply downstream of canonical ATFS-1 regulation.

## 4. Materials and Methods

### 4.1. C. elegans Strains

The following *C. elegans* strains were obtained from the Caenorhabditis Genetics Center (CGC): MQ887 (*isp-1(qm150)*), SJ4100 (zcIs13[*hsp-6p::GFP*]), EG7828(oxTi310 [*eft-3p::tdTomato::H2B*]), SJ4197 (zcIs39[*dve-1p::dve-1::gfp*]).

The following transgenic strains were generated in this study: MAT278 (jefEs75[*rpl-28p::gldi-8::flag::unc-54 3’UTR; myo-2p::mCherry::unc-54 3’UTR*]), MAT279 (jefIs30[*epc-1p::gfp::flag::unc-54 3’UTR; myo-2p::mCherry::unc-54 3’UTR*]), MAT280 (jefIs31[*epc-1p::gfp::flag::unc-54 3’UTR; myo-2p::mCherry::unc-54 3’UTR*]), MAT281 (jefIs32[*epc-1p::gfp::flag::unc-54 3’UTR; myo-2p::mCherry::unc-54 3’UTR*]), MAT282 (jefIs33[*epc-1p::gldi-8::gfp::flag::unc-54 3’UTR; myo-2p::mCherry::unc-54 3’UTR*].

Transgenic animals carrying extrachromosomal arrays were generated by germline microinjection using standard procedures. Injection mixtures generally contained 25 ng/μL of the plasmid of interest together with 5 ng/μL of *myo-2p::mCherry* as a co-injection marker. To establish stable integrated lines, extrachromosomal arrays were chromosomally integrated by X-ray irradiation, and the resulting strains were backcrossed with N2 at least five times before subsequent analysis.

### 4.2. RNA Interference

RNA interference (RNAi) was performed by bacterial feeding using HT115(DE3) bacteria carrying RNAi constructs from the Ahringer or Vidal library. Bacteria were grown overnight in LB medium containing 100 μg/mL ampicillin at 37 °C, seeded onto NGM plates supplemented with 1 mM IPTG, and induced at room temperature for 5 days before use. For RNAi experiments, synchronized worms were transferred onto RNAi plates at the L1 stage and maintained at 20 °C unless otherwise specified. Empty vector L4440 was used as the control (EV). For double-RNAi experiments, bacteria expressing the indicated RNAi constructs were mixed at a 1:1 ratio unless otherwise noted.

### 4.3. RNAi Screen for Nuclear Factors Required for UPR^mt^ Activation

RNAi-based screening was performed using a transcription factor RNAi sublibrary assembled by selecting transcription factor-targeting clones from the Ahringer and Vidal RNAi libraries. The screen was performed in worms carrying *isp-1(qm150)* and the integrated *hsp-6p::gfp* reporter. Synchronized animals were subjected to individual RNAi treatments, and GFP fluorescence was scored for suppression relative to empty vector controls. This screen identified *gldi-8* as a reproducible factor required for full activation of the *hsp-6p::gfp* reporter in response to mitochondrial stress.

### 4.4. UPR^mt^ Induction

Mitochondrial stress was induced either genetically or by RNAi-mediated knockdown of ETC genes. For genetic induction, worms carrying the *isp-1(qm150)* mutation were used as a constitutive mitochondrial stress model. For RNAi-induced stress, synchronized worms carrying the *hsp-6p::gfp* reporter were fed bacteria expressing *cco-1* dsRNA beginning at the L1 stage and analyzed after 3 days of treatment.

UPR^mt^ activation was assessed using the integrated reporter *hsp-6p::gfp*. Reporter induction was evaluated by fluorescence microscopy and, where indicated, quantified as described below.

### 4.5. Plasmid Construction and Transgenic Rescue

To generate the GLDI-8 translational reporter, the 2.3 kb upstream region of *epc-1* was amplified from genomic DNA and used to drive expression of *gldi-8::gfp*. The *epc-1* upstream region was selected because *epc-1* and *gldi-8* reside in the same operon. For promoter reporter analysis, the same *epc-1* upstream fragment was used to drive GFP expression alone.

For rescue experiments under *gldi-8* RNAi conditions, a codon-optimized gldi-8 coding sequence resistant to *gldi-8* RNAi was synthesized and placed under the control of the ubiquitous *rpl-28* promoter. Transgenic animals were generated by microinjection at 25 ng/μL together with co-injection markers *myo-2p::mCherry*.

### 4.6. Microscopy

For analysis of GLDI-8 localization, intestinal nuclei were identified using the nuclear marker *tdTomato::H2B* transgene. Worms were mounted on 2% agarose pads in M9 buffer containing 100 µg/mL levamisole. Nuclear accumulation of *GLDI-8::GFP* was assessed in worms exposed to EV or *cco-1* RNAi. Fluorescence imaging was performed using a Zeiss LSM800 confocal microscope (Carl Zeiss Microscopy GmbH, Jena, Germany) equipped with a 20× objective.

For promoter reporter analysis, worms carrying the *epc-1p::gfp* and *hsp-6p::gfp* reporters were anesthetized with M9 buffer containing 100 µg/mL levamisole and imaged using a Nikon SMZ18 stereomicroscope (Nikon Corporation, Tokyo, Japan), and the mean GFP fluorescence intensity was quantified using ImageJ software version 1.54g.

## Data Availability

The original contributions presented in this study are included in the article. Further inquiries can be directed to the corresponding author.
